# Why all newborn hip screening programs have same results—a mini review

**DOI:** 10.1007/s00431-024-05539-x

**Published:** 2024-04-09

**Authors:** Matias Vaajala BM, Oskari Pakarinen, Ilkka Helenius, Mikko M Uimonen, Ville T Ponkilainen, Ilari Kuitunen

**Affiliations:** 1 Faculty of Medicine and Life Sciences, University of Tampere, Tampere, Finland; 2 Department of Orthopaedics, New Childrens Hospital, Helsinki University Hospital, Helsinki, Finland; 3 Department of Orthopaedics, Faculty of Medicine, University of Helsinki, Helsinki, Finland; 4 Department of Surgery, Central Finland Central Hospital Nova, Jyväskylä, Finland; 5 Department of Pediatrics, Kuopio University Hospital, Kuopio, Finland; 6 Institute of Clinical Medicine, Department of Pediatrics, University of Eastern Finland, Kuopio, Finland

**Keywords:** Developmental dysplasia of the hip, Newborns, Universal ultrasound

## Abstract

All newborns are screened for developmental dysplasia of the hip (DDH), but countries have varying screening practices. The aim of this narrative mini review is to discuss the controversies of the screening and why it seems that all screening programs are likely to have same outcome. Different screening strategies are discussed alongside with other factors influencing DDH in this review. Universal ultrasound (US) has been praised as it finds more immature hips than clinical examination, but it has not been proven to reduce the rates of late-detected DDH or surgical management. Universal US screening increases initial treatment rates, while selective US and clinical screening have similar outcomes regarding late detection rates than universal US. This can be explained by the extrinsic factor affecting the development of the hip joint after birth and thus initial screening during the early weeks cannot find these cases.

*  Conclusion*: It seems that DDH screening strategies have strengths and limitations without notable differences in the most severe outcomes (late-detected cases requiring operative treatment). Thus, it is important to acknowledge that the used screening policy is a combination of values and available resources rather than a decision based on clear evidence.

## Developmental dysplasia of the hip

Developmental dysplasia of the hip (DDH) is an umbrella term used in the literature to describe several newborn hip conditions [1]. It includes early congenital dislocations, subluxations, loose hips, and in some cases clicky hips [1]. The total pooled incidence estimates for early detected DDH were 23.0 per 1000 newborns among those with universal ultrasonographic screening, 4.4 per 1000 among those with selective ultrasonographic screening, and 8.4 per 1000 newborns with clinical screening [2]. Typically, the newborn hips have been examined before discharge from the delivery hospital [3], yet there are many different screening strategies, and practices vary nationally and globally. In literature, the DDH is typically divided into early cases and late-detected cases [4]. The typical cutoff for late detected has varied between 3 and 6 months [4, 5]. The late-detected cases require more intensive treatment than early cases, i.e., closed reduction and longer splinting or harness treatment is needed and late-detected cases are more often treated operatively in open reductions or osteotomies [6, 7]. 

Untreated DDH leads to difficulties with moving and later promotes functional disability and early osteoarthritis [8]. Therefore, the commonly accepted goal has been to recognize the DDH cases as early as possible [9, 10]. The aim of screening practice is to be effective in terms of clinical findings and economically. However, with DDH this has not been the case, as the universal ultrasonography (US) screening programs have not been shown more effective or cost-effective than selective or clinical screening programs [11–13]. 

In this narrative mini review, we aim to synthesize and describe why newborn DDH screening programs are unlikely to succeed perfectly in practice.

## How are the newborn hips examined?

Clinical examination of the hip is performed by Ortolani and Barlow maneuvers which then are combined with clinical inspection of the asymmetry in the thighs and Galeazzi sign [14]. The most used US methods for hip evaluation are dynamic US and Graf US [15]. In the dynamic US, a posterior force is applied on the hip and the movement of the hip within the acetabulum or the change in the percentage cover can be measured. In the Graf US, the hip angles are measured, and the finding is then classified according to the scale (Table 1). The association between clinical screening findings and US findings has been weak [16]. Different countries have varying patterns on how many times the hips are clinically examined, what ages the possible US examinations are performed, and what are the criteria for selective US.

**Table 1:** Simplified classification scale of developmental dysplasia of the hip (DDH) based on the alpha-angle, which is a measurement of the bony roof of the acetabulum in the Graf ultrasonography

Type of DDH	Degree	Definition	Age
Type I	α ≥ 60°	Mature hip	All
Type II			
IIa	α 50–59°	Appropriate for age	< 3 months
IIb	α 50–59°	Inappropriate for age	> 3 months
IIc	α 43–49°		All
IId	α 43–49°	Decentring hip	
Type III	< 43°	Eccentric hip	All
Type IV	< 43°	Inverted labrum	All

## Natural progression of DDH

As stated earlier, the term DDH has been used to describe a variety of hip conditions. Traditionally, it has been assumed that the development of prenatally dysplastic or luxated hip joint may be directed towards a normal hip joint development with early diagnosis and appropriate interventions after birth. The need of interventions is determined according to the initial hip joint condition assessed using clinical and/or ultrasound examination. This developmental model assumes that early started treatment leads to normalizing of pathological hips while normal hips at the beginning continue normal development. In reality, this would mean that universal US screening programs detect abnormal cases most efficiently and, thereby, have the lowest late-detection rates. However, according to previous literature, it seems that this is not the case. Indeed, the incidence of DDH has been found to be up to 69.5 per 1000 newborns at the US screening while only 4.8 per 1000 newborns requiring treatment, yielding that a majority of ultrasound-examined hips diagnosed as pathological will mature towards normal during the first weeks of life [17]. Initially normal hips developed into normal hips, and no additional instances of DDH were found on follow-up [17]. Thus, universal screening results into overtreatment of hips which would mature normally without any treatment. On the other hand, it has also been shown that some hips seem normal in the initial US examination but later require operative treatment despite the universal ultrasound screening [18]. These findings challenge the traditional linear pathophysiological model of hip joint development. Instead, these findings suggest that also extrinsic factors have an effect on hip development after birth [19]. 

## Factors associated to DDH

There are numerous factors which are found to be associated with DDH in previous literature. Breech presentation, female sex, a positive family history, oligohydramnios, postmaturity, high birthweight, and older maternal age, and swaddling of the baby are known to be associated to DDH [20–23]. Other, less widely accepted risk factors are found to be multiple pregnancy, foot deformities, and torticollis [24, 25]. On the contrary, back-carrying has been connected to a lower incidence of DDH [26]. In addition, it has been found that cold weather is a possible risk factor for DDH [27]. The exact reason for this remains unknown, but tighter clothing or increased swaddling to protect the baby from the cold resulting in extension and adduction of the hips has been hypothesized as an explanation [27]. 

## Costs, effectiveness and health care resources

In relation to clinical screening, US screening is an expensive process [28, 29]. However with the limited benefit of US screening of DDH, it could be used in high-risk areas or in areas with sufficient resources in health care but should not be globally recommendable. Before implementation of universal US to wider use, a more rigorous investigation of its benefits is required, as currently, the evidence does not promote the use of it. This can be explained by the extrinsic factor affecting the development of the hip joint after birth and thus initial screening during the early weeks cannot find these cases [17, 18]. Also, the late detection and operative treatment rates with universal screening were similar to those among selectively and clinically screened newborns [2]. In addition, previous literature suggests, that the performance of DDH US screening examiners is highly related to the examiners’ experience, the US screeners should be highly trained to gain the potential benefit from the screening [30], which is a challenging combination within countries with limited resources in the health care and education.

## Conclusions

Hip screening strategies that focus on the first week or first month of life are likely to miss those cases that would develop into pathological hips due to extrinsic factors. Therefore, universal screening of DDH, especially using highly resource-consuming US, is a problematic concept in health care with limited resources. The possible US screening of DDH should only be focused on high-risk areas. The clinical examination and further examination with US could be addressed to newborns with known clinical risk factors. As the universal screening of DDH appears to be a truly challenging concept, future studies should focus on better recognizing the risk factors and creating universal guidelines for the optimal targeted screening of newborns at high risk for DDH (Table 2).

**Table 2:** Considerations and recommendations on future studies

	Issues	Suggestions
Study design	RCTs are unlikely to be conducted due to the large number of needed patients.	Quasi-experimental well-designed studies, for example prospectively considered policy change implementation studies.
Study size	Majority of prior studies have been too small to report precise estimates.	Utilize nationwide design or multinational collaborations to achieve enough participants and number of diagnoses
Diagnosis definitions of early detected DDH	All cases regardless of the clinical findings combined.	Better stratification between the cases to at least subluxated and complete dislocation.
Diagnosis definitions of late-detected DDH	Heterogeneous description of late-detected DDH as varying a timeline from two months to 12 months of age.	More use of continuous age reporting at the time of the DDH diagnosis to provide a better estimation of variation in age.
Study setting	Too vague description of the screening process and how the screening results are handled.	Exact description of the screening protocol and clear definitions of treatments were given.
Risk factor studies	Implementing risk factors without possible causality to DDH diagnosis to models and adjustments causes bias in estimations.	Presenting the causal pathways for potential risk factors in the analyses by for example using directed acyclic graphs.
Costs	Typically, the studies have not reported the cost of ultrasound screening in their setting.	Exact reporting of the costs of the screening programs per individual newborn.

## Data Availability

No datasets were generated or analyzed during the current study.

## References

[1] Nandhagopal T, De Cicco FL (2022) Developmental Dysplasia Of The Hip. In: *StatPearls*. StatPearls Publishing; Accessed 2 Feb 2023. http://www.ncbi.nlm.nih.gov/books/NBK563157/33085304

[2] Kuitunen I, Uimonen MM, Haapanen M, Sund R, Helenius I, Ponkilainen VT (2022). Incidence of neonatal developmental dysplasia of the hip and late detection rates based on screening strategy: a systematic review and Meta-analysis. JAMA Netw Open.

[3] Schwend RM, Schoenecker P, Richards BS, Flynn JM, Vitale M, Pediatric Orthopaedic Society of North America (2007). Screening the newborn for developmental dysplasia of the hip: now what do we do?. J Pediatr Orthop.

[4] Lehmann HP, Hinton R, Morello P, Santoli J (2000). Developmental dysplasia of the hip practice guideline: Technical report. Committee on Quality Improvement, and Subcommittee on Developmental Dysplasia of the Hip. Pediatrics.

[5] Guille JT, Pizzutillo PD, MacEwen GD (2000). Developmental dysplasia of the hip from birth to six months. JAAOS - J Am Acad Orthop Surg.

[6] Mankey MG, Arntz GT, Staheli LT (1993). Open reduction through a medial approach for congenital dislocation of the hip. A critical review of the Ludloff approach in sixty-six hips. J Bone Joint Surg Am.

[7] Terjesen T, Horn J (2020). Management of late-detected DDH in children under three years of age. Bone Joint Open.

[8] Barlow TG (1963). Early diagnosis and treatment of congenital dislocation of the hip. Proc R Soc Med.

[9] Shaw BA, Segal LS, Section on Orthopaedics (2016). Evaluation and referral for developmental dysplasia of the hip in infants. Pediatrics.

[10] Biedermann R, Riccabona J, Giesinger JM (2018). Results of universal ultrasound screening for developmental dysplasia of the hip: a prospective follow-up of 28 092 consecutive infants. Bone Joint J.

[11] Mahan ST, Katz JN, Kim YJ (2009). To screen or not to screen? A decision analysis of the utility of screening for developmental dysplasia of the hip. J Bone Joint Surg Am.

[12] Harper P, Gangadharan R, Poku D, Aarvold A (2020). Cost analysis of Screening programmes for developmental dysplasia of the hip: a systematic review. Indian J Orthop.

[13] Pandey RA, Johari AN (2021). Screening of newborns and infants for developmental dysplasia of the hip: a systematic review. Indian J Orthop.

[14] Aronsson DD, Goldberg MJ, Kling TF, Roy DR (1994). Developmental dysplasia of the hip. Pediatrics.

[15] Ömeroğlu H (2014). Use of ultrasonography in developmental dysplasia of the hip. J Child Orthop.

[16] Kyung BS, Lee SH, Jeong WK, Park SY (2016). Disparity between clinical and ultrasound examinations in neonatal hip screening. Clin Orthop Surg.

[17] Kokavec M, Bialik V (2007). Developmental dysplasia of the hip. Prevention and real incidence. Bratisl Lek Listy.

[18] Pan T, Armstrong DG, Hennrikus WL (2022). Late presenting developmental dysplasia of the hip after a normal hip ultrasound at 6 weeks of age: a report of two cases. J Paediatr Child Health.

[19] Rhodes AML, Clarke NMP (2014). A review of environmental factors implicated in human developmental dysplasia of the hip. J Child Orthop.

[20] de Hundt M, Vlemmix F, Bais JMJ (2012). Risk factors for developmental dysplasia of the hip: a meta-analysis. Eur J Obstet Gynecol Reprod Biol.

[21] Roposch A, Protopapa E, Malaga-Shaw O (2020). Predicting developmental dysplasia of the hip in at-risk newborns. BMC Musculoskelet Disord.

[22] Chan A, McCaul KA, Cundy PJ, Haan EA, Byron-Scott R (1997). Perinatal risk factors for developmental dysplasia of the hip. Arch Dis Child Fetal Neonatal Ed.

[23] Ulziibat M, Munkhuu B, Bataa AE, Schmid R, Baumann T, Essig S (2021). Traditional Mongolian swaddling and developmental dysplasia of the hip: a randomized controlled trial. BMC Pediatr.

[24] Xiao H, Tang Y, Su Y (2022). Risk factors of developmental dysplasia of the hip in a single clinical center. Sci Rep.

[25] Kural B, Devecioğlu Karapınar E, Yılmazbaş P, Eren T, Gökçay G (2019). Risk factor assessment and a ten-year experience of DDH screening in a well-child population. BioMed Res Int.

[26] Graham SM, Manara J, Chokotho L, Harrison WJ (2015). Back-carrying infants to prevent developmental hip dysplasia and its sequelae: is a new public health initiative needed?. J Pediatr Orthop.

[27] Loder RT, Shafer C (2014). Seasonal variation in children with developmental dysplasia of the hip. J Child Orthop.

[28] Schuler A, Reuss J, Delorme S, Hagendorff A, Giesel F (2010). [Costs of clinical ultrasound examinations-an economical cost calculation and analysis]. Ultraschall Med Stuttg Ger 1980.

[29] Shorter D, Hong T, Osborn DA (2013). Cochrane review: screening programmes for developmental dysplasia of the hip in newborn infants. Evid-Based Child Health Cochrane Rev J.

[30] Sewell MD, Eastwood DM (2011). Screening and treatment in developmental dysplasia of the hip—where do we go from here?. Int Orthop.

